# Floristic characteristics and affinities in Lao PDR, with a reference to the biogeography of the Indochina peninsula

**DOI:** 10.1371/journal.pone.0179966

**Published:** 2017-06-23

**Authors:** Hua Zhu

**Affiliations:** Center for Integrative Conservation, Xishuangbanna Tropical Botanical Garden, Chinese Academy of Sciences, Kunming, Yunnan, P. R. China; National Cheng Kung University, TAIWAN

## Abstract

The flora of Laos is composed of 5,005 species in 1,373 genera and 188 families of seed plants. Floristic and geographical attributes of the flora were analyzed. Tropical floristic elements at the family and generic levels contributed a majority (62.23% of the families and 82.30% of the genera) of the flora, of which the dominant geographical elements were pantropical distribution (42.02% of families) and tropical Asian distribution (30.08% of genera). This revealed that the flora of Lao PDR is tropical in nature and has a conspicuously tropical Asian affinity. Compared with the neighbouring countries of the Indochina peninsula, the flora of Laos has similar floristic composition and geographical elements. The floras of these Indochinese countries have similarities of more than 77.84% at the generic level, which suggests that they compose an affiliated biogeographical region. However, the flora of Laos showed the highest similarity to the flora of Vietnam (92.13%), followed by Myanmar (86.01%) at the generic level, but has less East Asian and North Temperate elements than Vietnam, and less North Temperate elements than Myanmar. These differences among the compared countries could be explained by the extrusion of the Indochinese block with the uplift of the Himalayas.

## Introduction

**Fig 1:**
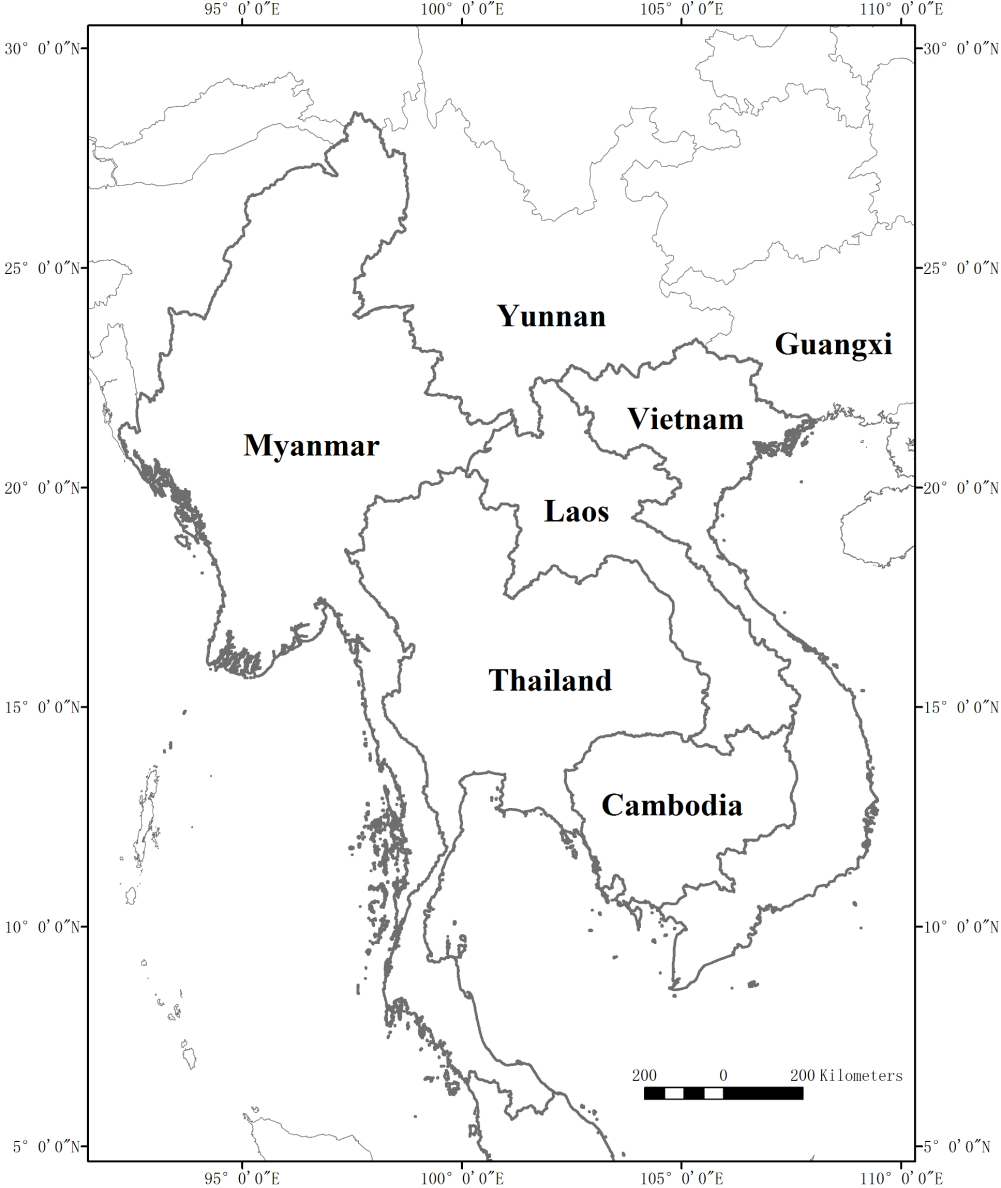
Geographical location of Lao PDR and the compared neighbouring countries. (The figure was made by the Landscape Ecology Lab., Xishuangbanna Tropical Botanical Garden, CAS).

**Fig 2:**
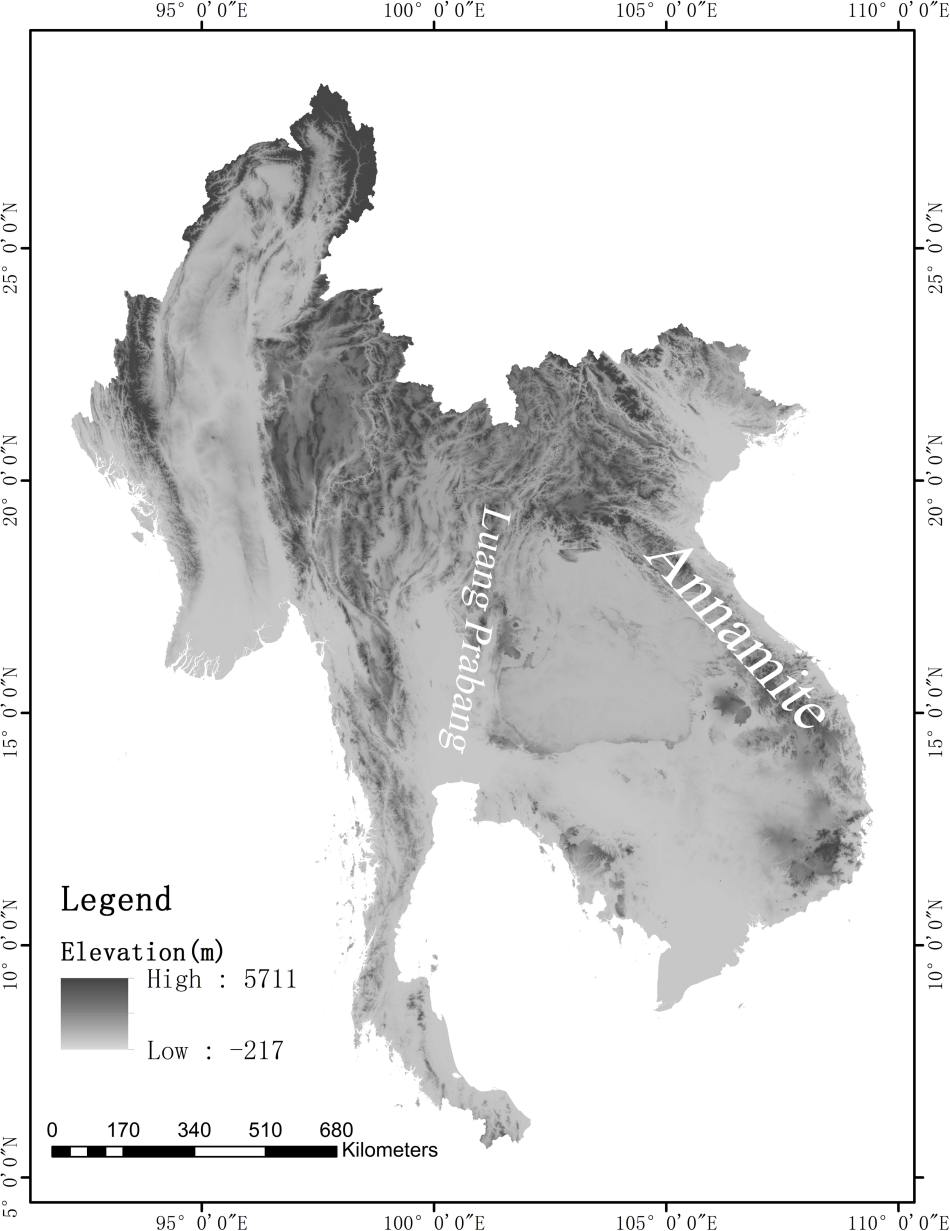
Topography of the Indochina peninsula. (The figure was made by the Landscape Ecology Lab., Xishuangbanna Tropical Botanical Garden, CAS).

Lao PDR (Lao People’s Democratic Republic), one of the world’s poorest countries, is located in the center of mainland Southeast Asia and covers 236,800 square kilometers (Fig 1). It is surrounded by Myanmar (Burma), Thailand, Cambodia, Vietnam, and the People's Republic of China. The topography of Laos is largely mountainous, with the Annamite Range in the northeast and east and the Luang Prabang Range in the northwest (Fig 2). It has elevations from 500 m in river valleys up to 2,819 m at the highest summit of Phou Bia mountain. Laos has a tropical monsoon climate, with a pronounced rainy season from May through October, a cool dry season from November through February, and a hot dry season in March and April [1].

Floristic and vegetation studies of Lao PDR are uncommon. Vidal [2, 3] described the forests of Laos. He distinguished the forest types of Laos as moist semi-deciduous forests with dipterocarps, mixed deciduous forests, and savanna in the lowlands, and moist montane forest of Fagaceae and Lauraceae species dominating the highlands. According to Blasco’s vegetation map of tropical continental Asia [4, 5], tropical lowland semi-evergreen rain forest, tropical dry deciduous forest and tropical moist deciduous forest, as well as dry deciduous dipterocarp woodland are the main vegetation types in the lowlands, and the tropical montane rain forest and gymnosperm forests at high elevations in Laos.

The flora of French Indochina began with a study from Loureiro [6], which was later revised by Lecomte et al. [7], and revised again by Aubrevile [8], in which the flora of Laos was included. There were two checklists referring to the plants of Lao PDR, i.e., Vidal [9] and Callanghan [10]. In Vidal’s list, more than 1,000 local names of plants were illustrated, and in Callanghan’s list more than 2,000 plant species were included. A new and updated checklist of the vascular plants of Lao PDR was recently published [11], in which 4,850 species including native, cultivated, and naturalized vascular plants were listed. Although the Newman's checklist is derived from a very incomplete data set, it is possible to use for a biogeographical analysis of Lao PDR.Understanding the floristic characteristics and affinities of Laos is important for understanding the plant geography of the Indochina peninsula. This paper aims to (i) analyze the floristic patterns and geographical elements of Laos; (ii) examine its floristic affinity to neighbouring countries; and (iii) discuss biogeography of the Indochina peninsula.

## Materials and methods

An updated list of native seed plants in Laos PDR is completed based upon entries in the checklist of the vascular plants of Lao PDR[11]; excluding cultivated, introduced, and invasive species and recent surveys conducted by Xishuangbanna Tropical Botanical Garden, Chinese Academy of Science. After compiling these entries, circumscription of families was updated following APG III [12, 13], and species names were rectified following the nomenclature and classification as presented in the w^3^TROPICOS of Missouri Botanical Garden (http://mobot.mobot.org/W3T/Search/vast.html). The updated list includes a total of 5,005 species in 1,373 genera and 188 families (S1 Table). Floristic and geographical attributes of the flora of Laos were analyzed based on the list. Patterns of seed plant distribution were quantified at the generic level mainly based on Wu’s documentation [14] and Wu et al. [15], Mabberley's Plant-Book [16], and at the family level following Wu et al.[17]. A minority of Wu’s circumscription on families did not corresponded with APG III. The distribution types of these variant families were determined by the actual distribution of these families. The geographical elements based on distribution types at family and generic levels were classified as: Cosmopolitan, Pantropic, Tropical Asia and Tropical America disjunct, Old World Tropics, Tropical Asia to Tropical Australia, Tropical Asia to Tropical Africa, Tropical Asia, North Temperate, East Asia and North America disjunct, Old World Temperate, Temperate Asia, Mediterranean region, West to Central Asia, Central Asia, East Asia and SW China to N Indochina. Comparisons of both floristic and geographical elements were made to assess the floristic similarity and variation as well as biogeographical affinities between neighbouring countries. Analyses were based upon published plant lists from Myanmar [18], Thailand [19], and Vietnam [20], and circumscription of families, genera, and species in these lists were rectified following APG III and the w^3^TROPICOS of Missouri Botanical Garden.

## Results

### Floristic composition

A total of 5,005 species in 1,373 genera and 188 families of native seed plants from Lao PDR were confirmed in total. Families with more than 100 species include Fabaceae (100 genera /521 species), Orchidaceae (96 /506), Euphorbiaceae (48/231), Rubiaceae (64/229), Poaceae (78/177), Lamiaceae (38/149), Cyperaceae (19/114), Fagaceae (4/109), Moraceae (7/108), Asteraceae (59/180), and Zingiberaceae (15/104) (Table 1). Of them, Orchidaceae, Euphorbiaceae, and Moraceae have a pantropic distribution. Zingiberaceae has a tropical Asian to tropical Australian distribution, Fagaceae has a north temperate distribution, and all others have a cosmopolitan distribution.

**Table 1:** Dominant (top 30) families and genera in species richness with their distribution.

Family ranking by their species richness	No. of species in the flora	Distribution type*	Genera ranking by their species richness	No. of species in the flora	Distribution type*
Fabaceae	521	2	*Dendrobium*	92	5
Orchidaceae	506	1	*Ficus*	75	2
Euphorbiaceae	231	2	*Bulbophyllum*	65	2
Rubiaceae	229	1	*Lithocarpus*	50	9
Poaceae	177	1	*Desmodium*	41	9
Lamiaceae	149	1	*Bauhinia*	38	2
Cyperaceae	114	1	*Ardisia*	36	2
Fagaceae	109	8	*Diospyros*	32	2
Moraceae	108	2	*Syzygium*	32	4
Zingiberaceae	104	5	*Crotalaria*	31	2
Scrophulariaceae	89	1	*Dalbergia*	31	2
Araceae	84	2	*Fimbristylis*	31	2
Apocynaceae	79	2	*Quercus*	31	8
Asteraceae	79	1	*Calamus*	29	5
Annonaceae	77	2	*Symplocos*	27	2
Convolvulaceae	67	1	*Castanopsis*	27	7
Acanthaceae	65	2	*Dioscorea*	26	2
Arecaceae	64	2	*Lindernia*	26	2
Rosaceae	62	1	*Eria*	26	5
Myrsinaceae	60	2	*Cyperus*	25	1
Rutaceae	59	2	*Croton*	24	2
Sterculiaceae	56	2	*Ipomoea*	24	2
Asclepiadaceae	54	2	*Millettia*	24	2
Malvaceae	49	2	*Oldenlandia*	24	2
Meliaceae	47	2	*Habenaria*	24	8
Myrtaceae	44	2	*Smilax*	22	2
Sapindaceae	42	2	*Eriocaulon*	20	2
Theaceae	42	2	*Indigofera*	20	2
Lauraceae	41	2	*Globba*	20	5
Commelinaceae	40	2	*Carex*	19	1

*Distribution type: 1: cosmopolitan, 2: pantropic, 4 Old World Tropic, 5 tropical Asia to tropical Australia, 7 tropical Asia, 8 north temperate, 9 east Asia and north America disjunct

At the generic level, *Dendrobium*, with 92 species, is the most species-rich genus, followed by *Ficus* (75 species), *Bulbophyllum* (65), *Lithocarpus* (50), *Desmodium* (41), *Bauhinia* (38), *Ardisia* (36), *Diospyros* (32), *Syzygium* (32), *Crotalaria* (31), *Dalbergia* (31), *Fimbristylis* (31), and *Quercus* (31). Among these 13 species-rich genera, the major distribution type is pantropic, which include *Ficus*, *Bulbophyllum*, *Bauhinia*, *Ardisia*, and *Diospyros*. *Lithocarpus* and *Desmodium* have an East Asian and North American disjunct distribution, and *Syzygium* has an Old World Tropic distribution. *Dendrobium* has a tropical Asian to tropical Australian distribution, and *Quercus* has a north temperate distribution (Table 1).

### Geographical elements

In the flora of Lao PDR, families with a tropical distribution contribute 62.23% of the total families of the flora including those with a pantropical distribution (42.02%), such as Annonaceae, Anacardiaceae, Burseraceae, Combretaceae, Ebenaceae, Sapindaceae, and Sapotacea. Araliaceae, Aquifoliaceae, Elaeocarpaceae, and Styracaceae contribute to 7.45% of floral diversity in Laos and have a tropical Asian and tropical American distribution. Additionally, flora with a tropical Asian to tropical Australian distribution, such as Zingiberaceae, contributes 4.26%, and families occurring in tropical Asia, such as Carlemanniaceae, Crypteroniaceae, Mastixiaceae, Pentaphragmaceae, and Sladeniaceae contribute 3.19% of flora diversity. Families with mainly temperate distributions contribute 15.43% of the total flora including Caprifoliaceae, Liliaceae, Pinaceae, Betulaceae, Fagaceae, and Salicaceae. Families with a cosmopolitan distribution contribute 22.34% of the total flora (Table 2).

**Table 2:** Geographical elements of seed plants at the family level in the flora of Laos.

Geographical elements at family level	Number of family	%*
1 **Cosmopolitan**	**42**	**22.34**
2 Pantropic	79	42.02
3 Tropical Asia and Tropical America disjunct	14	7.45
4 Old World Tropic	6	3.19
5 Tropical Asia to Tropical Australia	8	4.26
6 Tropical Asia to Tropical Africa	4	2.13
7 Tropical Asia	6	3.19
**Tropical elements (types 2–7) in total**	**117**	**62.23**
8 North Temperate	19	10.11
9 East Asia and North America disjunct	8	4.26
10 East Asia	2	1.06
**Temperate elements (types 8–10) in total**	**29**	**15.43**
Total number of families	188	100

*The number of families in each geographical element/ the number of families of all geographical elements times 100.

Patterns of seed plant distribution of the flora at the generic level are enumerated in Table 3. The genera with tropical Asian distribution, such as *Aganosma*, *Alphonsea*, *Amoora*, *Chukrasia*, *Crypteronia*, *Knema*, *Mastixia*, *Mitrephora*, *Mycetia*, and *Pterospermum*, show the highest percentage among all distribution types, contributing 30.08% to the flora. Genera with pantropic distribution, such as *Ardisia*, *Bauhinia*, *Cryptocarya*, *Capparis*, *Croton*, *Dioscorea*, *Gnetum*, *Morinda*, *Marsdenia*, *Piper*, and *Uncaria*, contribute 19.81% to the flora of Laos. Genera with tropical Asian to tropical Australian distribution, contributing 11.51%, include *Argyreia*, *Dillenia*, *Lagerstroemia*, *Loesenneriella*, *Murray*, and *Toona*. The genera with Old World tropical distribution, such as *Barringtonia*, *Carallia*, *Canarium*, *Chasalia*, *Dracaena*, *Fissistigma*, *Pandanus*, and *Polyalthia*, contribute 9.69% of flora diversity. Genera with tropical Asian to tropical African distribution include *Anogeissus*, *Bombax*, *Flacourtia*, *Garcinia*, *Mitragyna*, and *Premna*. In total, genera of tropical distribution (types two–seven) comprise 82.30% of the total diversity of the Laotian flora. The genera with northern temperate distribution, such as *Artemisia*, *Betula*, *Carpinus*, *Cornus*, *Corydalis*, *Pinus*, *Salix*, and *Sorbus*, contribute 4.30% of the total genera. The genera with East Asian distributions, such as *Actinidia*, *Belamcandia*, *Aspidistra*, *Cephalotaxus*, *Choerospondia*, *Gardneria*, *Hovenia*, *Pegia*, *Skimmia*, *Stachyrus*, and *Pterocarya*, contribute 3.20% of the total genera. In total, genera with temperate distributions (types eight–14) contribute 16.68% of the total number, including five genera that have SW China to N Indochina distribution such as *Calcareoboea*, *Craspedolobium*, *Cunninghamia*, *Drepanostachyum*, and *Tapiscia* (Table 3). Only two genera are recorded as endemic to Laos, i.e., *Laosanthus* of Zingiberaceae and *Iodocephalus* of Asteraceae.

**Table 3:** Geographical elements of seed plants at the generic level in the flora of Laos.

Geographical elements at generic level	Number of genera	%*
**1 Cosmopolitan**	59	4.30
2 Pantropic	272	19.81
3 Tropical Asia and Tropical America disjunct	48	3.50
4 Old World Tropic	133	9.69
5 Tropical Asia to Tropical Australia	158	11.51
6 Tropical Asia to Tropical Africa	106	7.72
7 Tropical Asia	413	30.08
**Tropical elements (types 2–7) in total**	**(1130)**	**(82.30)**
8 North Temperate	59	4.30
9 East Asia and North America disjunct	37	2.69
10 Old World Temperate	26	1.89
11 Temperate Asia	2	0.15
12 Mediterranean, W Asia to C Asia	9	0.66
13 East Asia	44	3.20
14 SW China to N Indochina	5	0.36
**Temperate elements (types 8–14) in total**	**(186)**	**(16.68)**
15 Endemic to Laos	2	0.15
Total number of genera	1373	100.00

*The number of genera in each geographical element/ the number of genera of all geographical elements times 100.

### Comparison with the floras of Vietnam, Thailand, and Myanmar

The floristic similarities at the generic and species levels between the floras of Laos, Vietnam, Thailand, and Myanmar are given in Table 4. The floristic similarities between the flora of Laos and the floras from neighbouring countries exceed 77.84% at the generic level. The flora of Laos shows the highest similarity to the flora of Vietnam (92.13%), followed by the flora of Myanmar (86.01%). However, at specific level, except for the similarity between Laos and Vietnam, which is 61.19%, all other countries have relatively low similarities (lower than 45%). The lowest similarity at the specific level exists between Vietnam and Myanmar.

**Table 4:** Comparison of floristic similarities at the generic and specific levels.

Compared flora	Laos	Vietnam	Thailand	Myanmar	
	Similarity coefficient (%)	Similarity coefficient (%)	Similarity coefficient (%)	Similarity coefficient (%)	
Similarity coefficients at generic level*					
Laos	100				
Vietnam	92.13	100			
Thailand	77.84	87.53	100		
Myanmar	86.01	79.93	83.93	100	
Similarity coefficients at specific level*					
Laos	100				
Vietnam	61.19	100			
Thailand	42.47	44.8	100		
Myanmar	40.07	32.49	39.18	100	

*Similarity coefficient between A and B = the number of taxa shared by both A and B divided by the lowest number of taxa of A or B, multiplied by 100%

The comparison of geographical elements at the family level shows that these compared floras are almost the same. Laos and its neighbouring countries have tropical families contributing to 59.82%– 62.23% and temperate families contributing 15.43%– 19.57% of floral diversity. The minor differences are: the north temperate and East Asian families are more often found in Myanmar and the highest ratio of temperate families is found in Vietnam (Table 5).

**Table 5:** Comparison of geographical elements of seed plants at the family level.

Compared regional floras	Laos (188 families)	Vietnam (231 families)	Thailand (201 families)	Myanmar (209 families)
Geographical elements at family level	%*	%*	%*	%*
**1 Cosmopolitan**	**22.34**	**20.00**	**22.39**	**22.49**
2 Pantropic	42.02	38.26	41.79	38.76
3 Tropical Asia and Tropical America disjunct	7.45	6.52	5.47	6.22
4 Old World Tropic	3.19	4.35	4.98	4.31
5 Tropical Asia to Tropical Australia	4.26	3.91	3.48	3.83
6 Tropical Asia to Tropical Africa	2.13	2.61	1.99	2.87
7 Tropical Asia	3.19	4.78	3.98	3.83
**Tropical elements (types 2–7) in total**	**62.23**	**60.43**	**61.69**	**59.82**
8 North Temperate	10.11	12.17	10.45	14.35
9 East Asia and North America disjunct	4.26	4.35	3.48	0.00
10 Old World Temperate	0	0.43	0.00	0.00
Mediterranean, W Asia to C Asia	0	0.43	0.00	0.00
11 East Asia	1.06	2.17	1.99	3.35
**Temperate elements (types 8–11) in total**	**15.43**	19.57	**15.92**	**17.7**
Total	188	230	201	209

*The number of family in each geographical element/ the number of family of all geographical elements times 100.

The comparison of geographical elements at the generic level from these regional floras shows some differences. Although the tropical elements (types two–seven) contribute more than 72.52% of the total genera in all of the compared floras, the highest proportion of the tropical elements occurs in the flora of Thailand (comprising 84.95% of all the genera). The flora of Myanmar has a higher proportion of geographical elements of temperate flora (22.75%), such as north temperate and east Asia elements (Table 6).

**Table 6:** Comparison of geographical elements of seed plants at the generic level.

Compared regional floras	Laos (1373 genera)	Vietnam (2017 genera)	Thailand (1475 genera)	Myanma (1903)
Geographical elements at generic level	%*	%*	%*	%*
1 Cosmopolitan	4.30	3.87	3.86	4.47
2 Pantropic	19.81	17.60	20.88	17.66
3 Tropical Asia and Tropical America disjunct	3.50	3.27	2.31	3.10
4 Old World Tropic	9.69	9.07	10.64	8.99
5 Tropical Asia to Tropical Australia	11.51	9.77	11.66	9.56
6 Tropical Asia to Tropical Africa	7.72	6.74	7.32	6.73
7 Tropical Asia	30.08	29.65	32.14	26.48
**Tropical elements (types 2–7) in total**	**82.30**	**76.10**	**84.95**	**72.52**
8 North Temperate	4.30	6.30	3.93	7.46
9 East Asia and North America disjunct	2.69	2.83	1.83	3.05
10 Old World Temperate	1.89	2.68	1.42	3.73
11 Temperate Asia	0.15	0.35	0.14	0.37
12 Mediterranean, W Asia to C Asia	0.66	0.79	0.54	1.26
13 Center Asia	3.20	0.05	0.07	0.32
14 East Asia	0.36	5.85	2.78	6.20
15 SW China to N Indochina	4.30	1.19	0.07	0.37
**Temperate elements (types 8–15) in total**	**13.26**	**20.03**	**10.78**	**22.75**
Endemic to Laos	0.15	0	0	
Endemic to Vietnam		0	0	
Endemic to Burma			0.41	
Endemic to Burma				0.26
Total	100.00	100.00	100.00	100.00

*The number of genera in each geographical element/ the number of genera of all geographical elements times 100.

## Discussion

Like the floras of southern China from the northern margin of tropical Asia [21–25], the flora of Lao PDR and other countries in Indochina peninsula, have a similar floristic composition and tropical Asian affinity, The flora of Laos is dominated by the same species-rich families and genera such as Fabaceae, Orchidaceae, Euphorbiaceae, Rubiaceae, Poaceae, Lamiaceae, Fagaceae, Moraceae, Zingiberaceae, *Ficus*, *Bulbophyllum*, *Lithocarpus*, *Desmodium*, *Bauhinia*, *Ardisia*, *Diospyros*, *Syzygium*, and *Dalbergia*. It also has a majority of tropical elements (62.23% of the total families and 82.30% of the total genera). The most dominant genera are those from tropical Asia (30.08% of the total genera). This reveals that the flora of Lao PDR is tropical in nature and has a conspicuously tropical Asian affinity.

**Fig 3:**
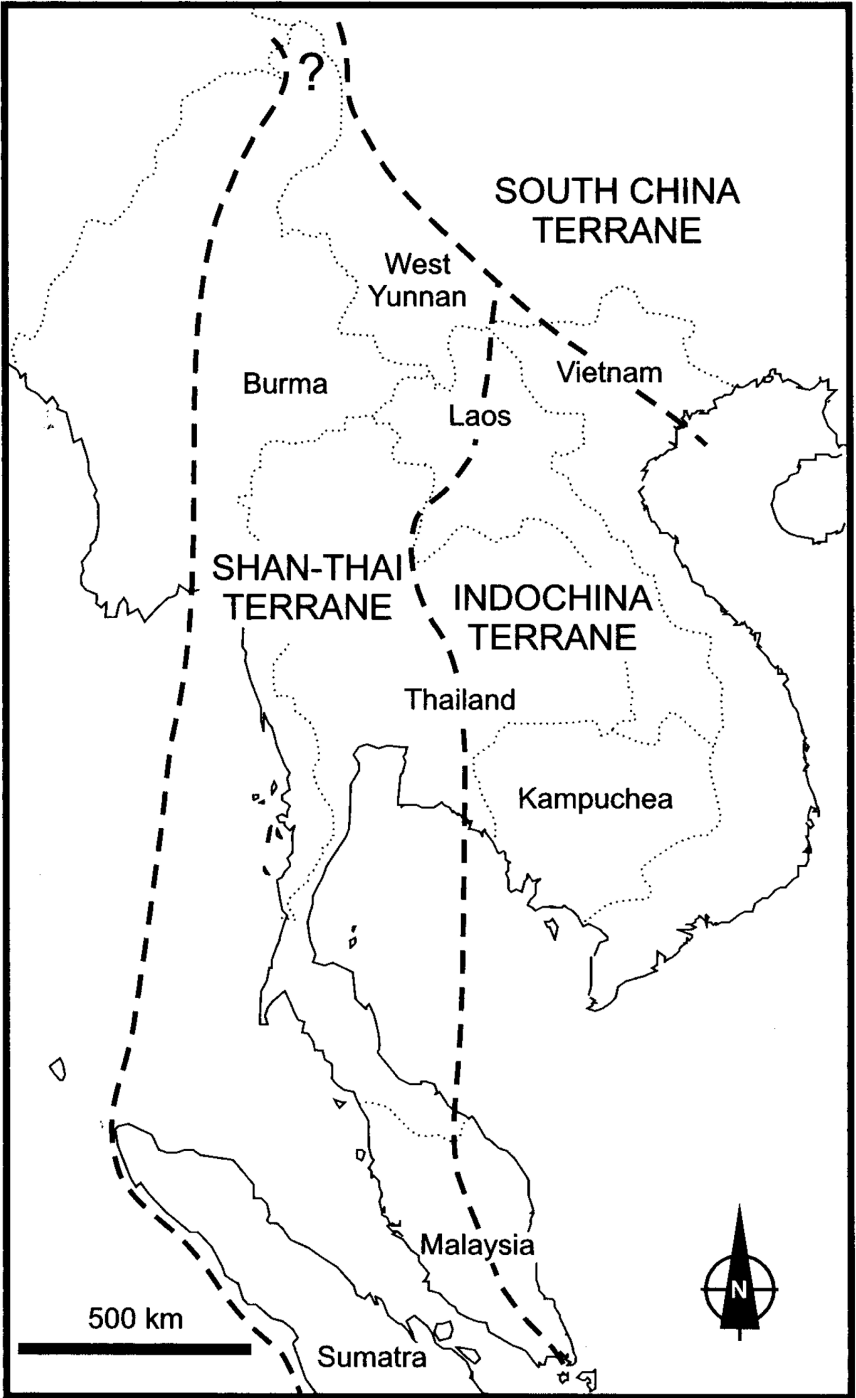
The main geoblocks in Southeast Asia (from Fortey et al., 1998).

Located at the centre of the Indochina peninsula, the flora of Laos is similar to the floras of Vietnam, Thailand, and Myanmar at the family and genera level (for example, more than 77.84% of similarity at the generic level). Additionally, Laos and its neighbouring countries have a similar composition in geographical elements. Our research has shown that the counties of the Indochina peninsula are floristically continuous and compose an affiliated biogeographical region. Moreover, the flora of Laos has the highest similarity to the flora of Vietnam (92.13% at the generic level and 61.19% at the specific level). It is logical that Laos and Vietnam have the most similar flora because of the physical geographic connection between them. Additionally, Vietnam has more East Asian and north temperate elements than the flora of Laos. Compared with the other neighbouring countries, the flora of Myanmar has the lowest proportion in tropical elements and the highest in temperate elements, while the flora of Thailand has the highest tropical elements and the lowest temperate elements. The biogeography of the Indochina peninsula could be explained by geologic block movements with the uplift of the Himalayas in these regions [26–32]. Fig 3 shows the boundaries of the main geoblocks in Southeast Asia [33]. Continental Southeast Asia comprises a collage of blocks bounded by suture zones [32]. The Indochina peninsula was formed from the Indochina block or terrane, South China block, Shan-Thai terrane and West Burma block [33]. These geoblocks each have a different geological history. With the collision between India and Eurasia, which began in the early Cenozoic [34], the Indochina block extruded southeastward along the Ailao Shan-Red River shear zone in the Oligocene and early Miocene [26–28], while the Shan-Thai terrane underwent displacements and strike-slip faulting [35]. India collided with northern Myanmar and Tibet in the latest Eocene, and since the latest Cretaceous, the Burma plate has moved 1100 km northward relative to the Asian plate to the east [36]. The geological history of the Indochina blocks could have affected its biogeography.

Tropical Asian elements conspicuously influenced the flora of Laos during the extrusion of the Indochinese block (southward translocation of the Indochina Block) with uplift of the Himalayas. This could have resulted in the tropical Asian affinity of its flora. The flora of Vietnam has more east Asian and north temperate elements because the northeastern part of Vietnam is part of the East Asian geoblock. The flora of Myanmar has the lowest tropical elements and the highest in temperate elements, which could be because northern Myanmar is contiguous to the Himalayas and became influenced by the temperate flora of Himalayas.

## Conclusions

A revised list of native seed plants from Laos PDR is presented comprising 5,005 species in 1,373 genera and 188 families. Geographical elements show that the families with tropical distribution contribute 62.23% of the total sum of the flora including flora with pantropical distribution (42.02%). The genera with a tropical distribution contribute 82.30%, in which the genera of tropical Asia make up 30.08%. This reveals that the flora of Lao PDR is tropical in nature and has a conspicuously tropical Asian affinity. The flora of Laos has similar floristic composition and geographical elements to its neighbouring countries from the Indochina peninsula and belongs to the same biogeographical unit. Compared with other Indochinese countries, the flora of Laos has the highest similarity with the flora of Vietnam (92.13% at the generic level and 61.19% at the specific), followed by Myanmar at the generic level and Thailand at the specific level. However, the floras of Myanmar and Vietnam have more north temperate and east Asian elements. The biogeography of Indochinese countries could be explained not only by their geographical locations, but also by geologic block movements of these regions. Specifically, the extrusion of the Indochinese block took place with the uplift of the Himalayas.

## Supporting information

S1 Table Native seed plant list in Lao PDR. (The circumscriptions of families follow APG III, and species nomenclature follows w^3^TROPICOS (http://mobot.mobot.org/W3T/Search/vast.html).
(XLS)
