# Correction: Floristic characteristics and affinities in Lao PDR, with a reference to the biogeography of the Indochina peninsula

**DOI:** 10.1371/journal.pone.0184716

**Published:** 2017-09-07

**Authors:** Hua Zhu

There are errors in Table 6. In the first row, "Myanma" should be "Myanmar." In row 22, "Endemic to Burma" should be "Endemic to Thailand." In row 23, "Endemic to Burma" should be “Endemic to Myanmar.” Please see the corrected Table 6 here.

**Table 6:** Comparison of geographical elements of seed plants at the generic level.

Compared regional floras	Laos (1373 genera)	Vietnam (2017 genera)	Thailand (1475 genera)	Myanmar (1903)
Geographical elements at generic level	%*	%*	%*	%*
1 Cosmopolitan	4.30	3.87	3.86	4.47
2 Pantropic	19.81	17.60	20.88	17.66
3 Tropical Asia and Tropical America disjunct	3.50	3.27	2.31	3.10
4 Old World Tropic	9.69	9.07	10.64	8.99
5 Tropical Asia to Tropical Australia	11.51	9.77	11.66	9.56
6 Tropical Asia to Tropical Africa	7.72	6.74	7.32	6.73
7 Tropical Asia	30.08	29.65	32.14	26.48
**Tropical elements (types 2–7) in total**	**82.30**	**76.10**	**84.95**	**72.52**
8 North Temperate	4.30	6.30	3.93	7.46
9 East Asia and North America disjunct	2.69	2.83	1.83	3.05
10 Old World Temperate	1.89	2.68	1.42	3.73
11 Temperate Asia	0.15	0.35	0.14	0.37
12 Mediterranean, W Asia to C Asia	0.66	0.79	0.54	1.26
13 Center Asia	3.20	0.05	0.07	0.32
14 East Asia	0.36	5.85	2.78	6.20
15 SW China to N Indochina	4.30	1.19	0.07	0.37
**Temperate elements (types 8–15) in total**	**13.26**	**20.03**	**10.78**	**22.75**
Endemic to Laos	0.15	0	0	
Endemic to Vietnam		0	0	
Endemic to Thailand			0.41	
Endemic to Myanmar				0.26
Total	100.00	100.00	100.00	100.00

*The number of genera in each geographical element/ the number of genera of all geographical elements times 100.
